# Extensive Proximal Deep Vein Thrombosis Despite Normal Age-Adjusted D-Dimer Levels: A Case Report

**DOI:** 10.7759/cureus.98052

**Published:** 2025-11-28

**Authors:** Luigi Dimech, Ian Galea, Jonathan Calleja

**Affiliations:** 1 Geriatrics, Mater Dei Hospital, Msida, MLT; 2 Family and Community Medicine, Gozo General Hospital, Gozo, MLT; 3 Geriatrics, Gozo General Hospital, Gozo, MLT

**Keywords:** age-adjusted cutoffs, d-dimer, deep vein thrombosis (dvt), sensitivity, specificity

## Abstract

Deep vein thrombosis (DVT) refers to the formation of a blood clot within a deep vein, most often in the lower extremities, which can partially or completely obstruct venous blood flow. This case discusses a 93-year-old female nursing home resident who developed extensive left lower limb swelling with a laboratory D-dimer level of 316 ng/mL (normal range for a 93-year-old: 0-930 ng/mL). Based on her age-adjusted D-dimer threshold of 930 ng/mL, thrombosis would ordinarily be considered unlikely. However, given her predominantly wheelchair-bound status and a Wells score of 3, the clinical suspicion for DVT remained high. Doppler ultrasound subsequently confirmed extensive proximal thrombosis with complete occlusion of the left popliteal and femoral veins. This case illustrates the limitations of relying solely on D-dimer testing to exclude DVT in elderly patients and underscores the importance of clinical judgment.

## Introduction

Deep vein thrombosis (DVT) is characterised by the formation of a thrombus within the deep venous system, most commonly in the lower extremities [1]. Its pathophysiology is classically described by Virchow’s triad: venous stasis, often resulting from immobilisation or surgery; endothelial injury, typically due to trauma; and hypercoagulability, which may be associated with malignancy or inherited clotting factor mutations [2]. Additional risk factors include advanced age, obesity, smoking, infection, and a previous history of thromboembolic events [3]. 

The most serious acute complication of DVT is pulmonary embolism (PE), which can potentially lead to sudden death [4]. Long-term complications, particularly in patients with extensive proximal DVT, such as the present case, include post-thrombotic syndrome (PTS) [5]. Furthermore, individuals with a history of DVT are at increased risk of recurrence, with up to 30% experiencing another episode within ten years, especially if the initial event was unprovoked [6]. 

## Case presentation

Our 93-year-old female patient developed left lower limb swelling overnight, which extended from the foot up to the proximal thigh. There were no respiratory symptoms such as shortness of breath, chest pain, or haemoptysis to suggest PE. 

Past medical history 

Her past medical history included frailty, hypertension, hypercholesterolaemia, osteoporosis, stroke with no residual neurological deficits, falls and recurrent episodes of pseudo-obstruction. Recently, she had suffered from another bout of intestinal pseudo-obstruction, resulting in progressive immobility, transitioning from being wheelchair-bound to predominantly bedbound. 

Physical examination 

On examination, the patient was hemodynamically stable with a National Early Warning Score (NEWS) 2 score of 0 [7] and appeared in no acute distress. Heart sounds were normal, lung auscultation was unremarkable, and the abdomen was soft and non-tender. 

The left lower limb was diffusely swollen and mildly tender, with a circumference of 28 cm measured 10 cm below the tibial tuberosity, compared to 23 cm on the contralateral side. Bilateral pitting oedema was present, more marked on the affected limb. There were no overlying skin changes, ulcerations, or varicose veins. 

Diagnosis and management 

Initial workup included a D-dimer level of 316 ng/mL and a Wells score of 3 [8]. The Wells score result was based on recent findings, namely, immobilisation, entire leg swelling, and a calf circumference difference exceeding 3 cm. Empiric therapeutic anticoagulation was initiated with low molecular weight heparin (LMWH). Doppler ultrasound of the left lower extremity confirmed complete thrombosis of the popliteal and femoral veins, consistent with left extensive proximal DVT. 

After confirmation of the proximal DVT with sonography, she was switched from LMWH to a direct oral anticoagulant (DOAC). Supportive measures, such as gradual mobilisation, limb elevation, and compression therapy, were also commenced. 

**Figure 1:**
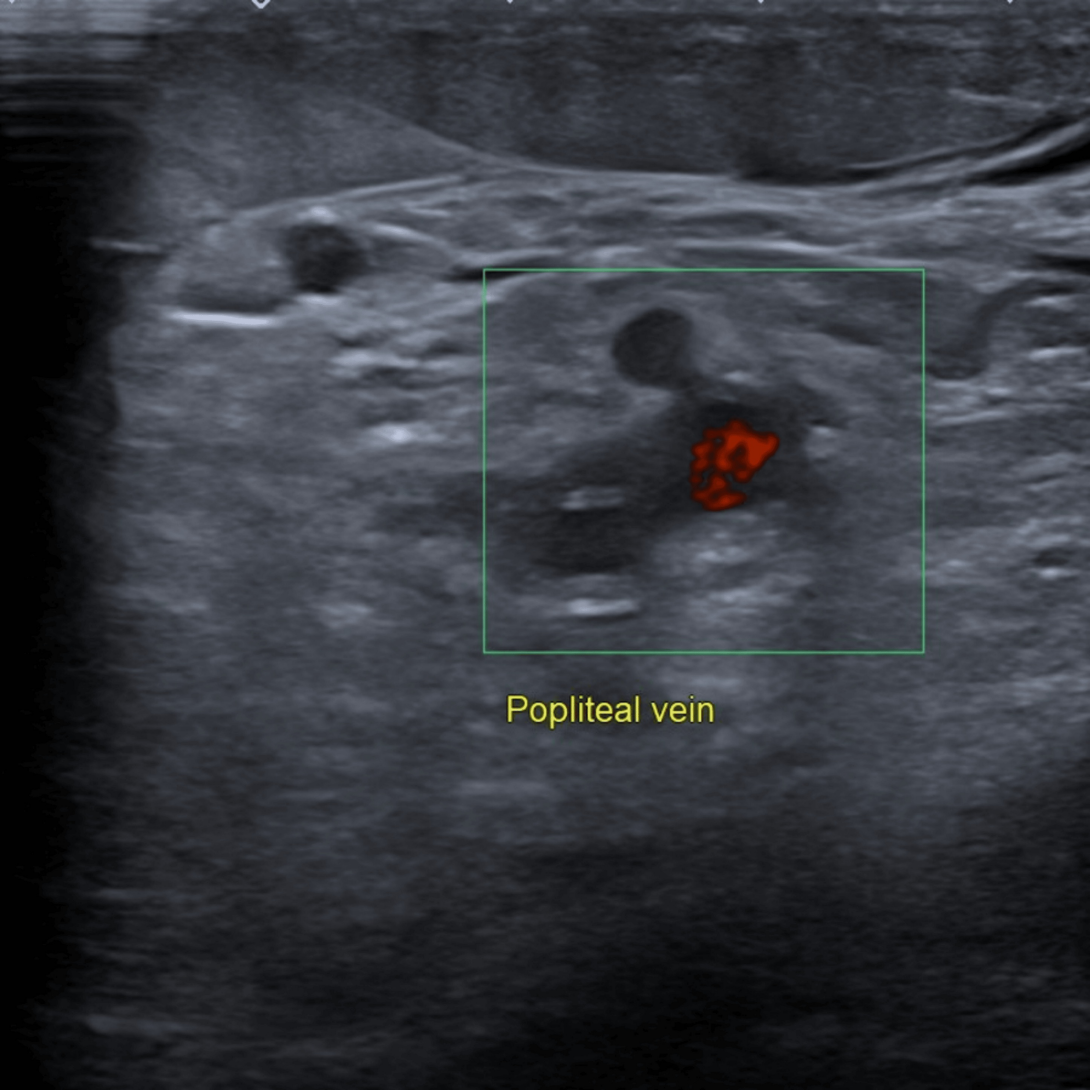
Left Popliteal Vein

**Figure 2:**
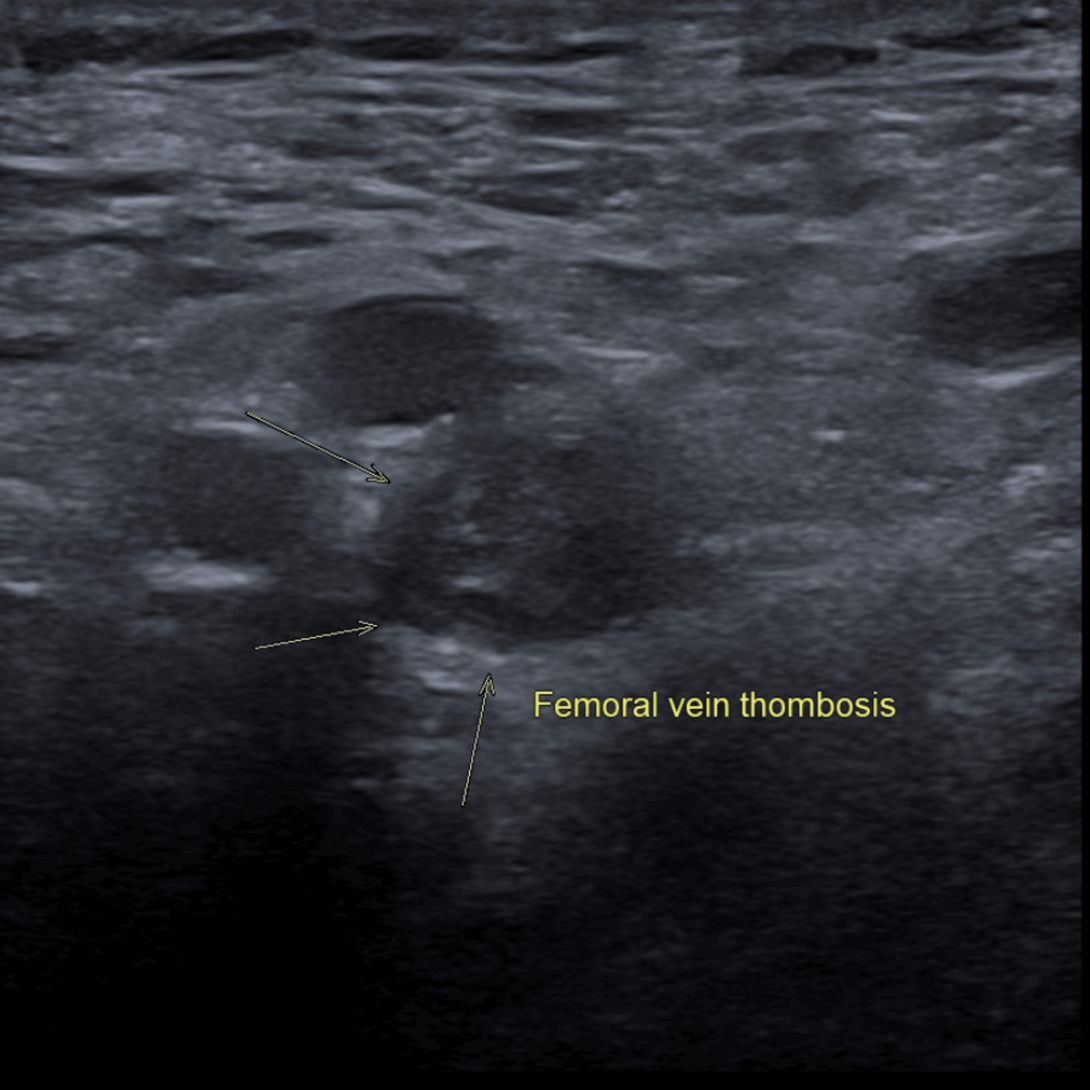
Sonographic Evidence of Femoral Vein Thrombosis

**Figure 3:**
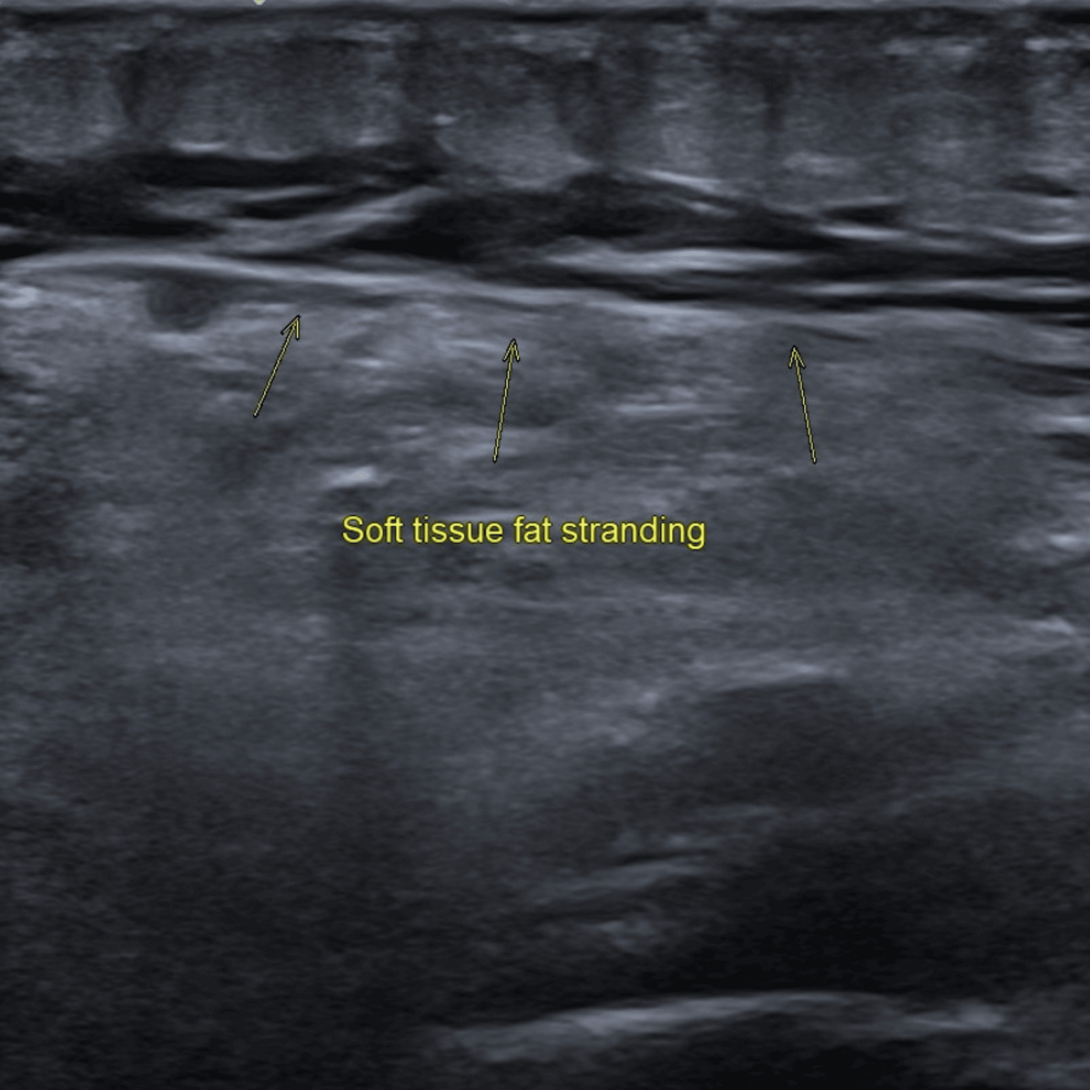
Soft Tissue Fat Stranding Around the Thrombosis

The following images illustrate the ultrasonographic findings consistent with proximal DVT: (1) Figure 1 displays the left popliteal vein highlighted in red, indicating blood flow directed toward the ultrasound probe; (2) Figure 2 reveals a non-compressible femoral vein with intraluminal echogenic material; these findings are indicative of extensive DVT; (3) Figure 3 demonstrates increased echogenicity of the surrounding soft tissue, consistent with fat stranding; these changes suggest localised inflammation associated with the adjacent thrombus.

## Discussion

DVT is a common vascular condition with significant morbidity and mortality, particularly in elderly and immobile patients. The diagnosis is often supported by a specific blood test marker called the D-dimer, due to its high sensitivity; however, specificity decreases with advancing age [9]. Age-adjusted D-dimer thresholds have been proposed to improve diagnostic accuracy in older patients [10]. This case highlights the diagnostic challenges posed by a subthreshold D-dimer level in a high-risk elderly home resident who turned out to have an extensive proximal DVT. 

D-dimer is a fibrin degradation product widely used as a sensitive biomarker to exclude venous thromboembolism (VTE) in patients with low to moderate pretest probability. The diagnostic performance of the D-dimer assay changes with age, as sensitivity and specificity are affected differently. Sensitivity, which is the ability to detect true positives, generally remains high even in older adults, making a negative result useful for ruling out venous thromboembolism [9]. However, our case is notable because, despite this high sensitivity, the test failed to identify a confirmed extensive proximal DVT, highlighting that false negatives, while uncommon in the elderly, can still occur. In contrast, specificity, which is the ability to correctly identify true negatives, declines markedly with age. Older individuals often have elevated baseline D-dimer levels due to increased fibrin turnover, chronic inflammation, subclinical disease, and reduced clearance, all of which contribute to a higher rate of false positives [11]. 

For individuals over the age of 50, the following formula is used [10]:

$\text{Age-adjusted D-dimer} = \text{Patient's age} \times 10\,\text{ng/mL}$

Using a fixed cut-off of 500 ng/mL in patients over 50 years frequently flags individuals without true thrombosis, leading to unnecessary imaging. To address this limitation, many guidelines now endorse age-adjusted D-dimer thresholds, which improve specificity without compromising sensitivity in older populations [11]. The most widely accepted method uses the aforementioned formula. In our patient, the threshold would be 930 ng/mL, making the measured value of 316 ng/mL appear falsely reassuring. 

Our case highlights that a low D-dimer level should not take precedence over clinical assessment, particularly in elderly individuals with a moderate to high pretest probability of DVT [11, 12]. Various factors, such as delayed presentation after symptom onset, prolonged immobility, and a diminished systemic inflammatory response, can result in deceptively low D-dimer levels even in the presence of significant thrombosis [13]. Moreover, the American Society of Haematology recommends that in patients with a Wells score of 2 or higher, D-dimer testing should not be used to exclude DVT. Instead, compression ultrasonography is advised as the preferred first-line diagnostic modality [12].

## Conclusions

This case highlights the diagnostic challenges encountered when evaluating venous thromboembolism in elderly patients, particularly when laboratory findings such as D-dimer levels appear incongruent with clinical presentation, despite age adjustment. It underscores the limitations of relying solely on laboratory diagnostics and highlights the necessity of a comprehensive approach that integrates clinical judgment and validated risk stratification tools, prior to therapeutic anticoagulation and definitive imaging. Such a multifaceted strategy is especially crucial in older adults, where comorbidities, altered physiology, and atypical presentations can complicate diagnosis. Ultimately, this case reinforces that optimal patient care relies on the integration of clinical assessment and diagnostic testing, rather than over-reliance on isolated biomarkers. In older, immobile patients, especially those with a moderate to high Wells score, a normal or low D-dimer level should not be considered sufficient to exclude DVT. 
